# Trends in Prevalence of Early Introduction of Complementary Foods to US Children, 2016 to 2022

**DOI:** 10.1001/jamanetworkopen.2024.40255

**Published:** 2024-10-11

**Authors:** Guodong Ding, Anqi Peng, Yan Chen, Angela Vinturache, Yongjun Zhang

**Affiliations:** 1 Department of Pediatrics, Xinhua Hospital, Shanghai Jiao Tong University School of Medicine, Shanghai, China; 2 Clinical Medical College, Yangzhou University, Yangzhou, Jiangsu Province, China; 3 Department of Obstetrics & Gynecology, University of Alberta, Edmonton, Alberta, Canada

## Abstract

This cross-sectional study analyzes temporal changes in prevalence of early complementary food introduction to US children from 2016 to 2022 and provides up-to-date evidence of this nutrition practice.

## Introduction

The American Academy of Pediatrics recommends introducing complementary foods to infants at 6 months.^1,2^ Although there is no consensus on ideal timing, most agree that introduction of complementary foods before 4 months is too early.^1,2^ Early introduction precludes the first 6 months of exclusive breastfeeding and may increase risk of childhood obesity.^3^ Previous studies reported that 1 in 6 US infants was introduced to complementary foods before 4 months.^4,5^ This report analyzes temporal changes in prevalence of early complementary food introduction to US children from 2016 to 2022 and provides up-to-date evidence.

## Methods

The US National Survey of Children’s Health (NSCH) is a representative survey for monitoring the health and well-being of noninstitutionalized children 17 years or younger.^6^ Data are collected annually from parents or other primary caregivers. This study used the NSCH data from 2016 to 2022 (released on April 24, 2024) to assess trends in prevalence of early introduction (<4 months) of complementary foods to children aged 1 year or younger. Parents or caregivers provided written informed consent for data collection. Because publicly available, anonymized data were used, the institutional review board of Xinhua Hospital, Shanghai Jiao Tong University School of Medicine, deemed this study exempt from review. This study followed the STROBE reporting guideline.

Timing of introduction of complementary foods was assessed by asking “How old was this child when he or she was first fed anything other than breast milk or formula?” The survey package in R statistical software, version 4.3.2, was used, taking into account the complex survey design and weights. Weighted data were used to assess sociodemographic characteristics and prevalence of early introduction of complementary foods. Trends and changes in slopes (up to 1 joinpoint) were assessed using Joinpoint, version 5.0.2, by calculating the mean annual percentage change and 95% CI, overall and among subgroups. A 2-tailed *P* < .05 indicates significance.

## Results

From 21 860 children, we excluded 566 with missing data on the start of complementary foods, 157 who started after 12 months, and those with implausible feeding patterns (recalled breastfeeding duration, infant formula introduction, and complementary feeding introduction indicated ≥2 months with no source of nutrition [n = 37]; answer for all 3 feeding patterns was “no” [n = 16]). We also excluded 2900 infants younger than 4 months, leaving 18 184 children (51.4% boys and 48.6% girls). Table 1 presents the study population’s characteristics.

**Table 1.** Unweighted Sample Sizes and Survey-Weighted Frequencies of US Infants Aged 4 Months to 1 Year From National Survey of Children’s Health, 2016 to 2022

Characteristic	No. of participants (survey-weighted %)^a^							
	2016 (n = 3358)	2017 (n = 1408)	2018 (n = 1866)	2019 (n = 1607)	2020 (n = 2528)	2021 (n = 3677)	2022 (n = 3740)	Overall (N = 18 184)
Race and ethnicity^b^								
Hispanic	366 (23.4)	158 (23.0)	236 (23.3)	202 (25.9)	355 (23.7)	511 (23.3)	616 (25.3)	2444 (24.0)
Non-Hispanic Black	142 (13.3)	60 (8.8)	120 (11.3)	90 (10.1)	142 (12.3)	195 (11.0)	174 (11.0)	923 (11.1)
Non-Hispanic White	2451 (52.7)	1001 (52.9)	1293 (53.6)	1106 (52.8)	1672 (52.0)	2478 (52.8)	2420 (50.3)	12 421 (52.5)
Other	399 (10.6)	189 (15.3)	217 (11.9)	209 (11.2)	359 (12.0)	493 (12.9)	530 (13.4)	2396 (12.5)
Family income, % federal poverty level^c^								
<100	339 (23.4)	168 (18.5)	240 (19.6)	201 (20.0)	346 (19.6)	454 (18.7)	460 (18.2)	2208 (19.7)
100-199	505 (19.1)	222 (20.5)	325 (22.2)	291 (20.1)	428 (19.2)	571 (18.4)	547 (17.8)	2889 (19.7)
200-399	1054 (27.4)	435 (28.8)	552 (26.1)	492 (24.4)	771 (26.9)	1120 (27.3)	1098 (26.6)	5522 (26.8)
≥400	1460 (30.1)	583 (32.2)	749 (32.1)	623 (35.5)	983 (34.3)	1532 (35.6)	1635 (37.4)	7565 (33.8)
Highest education of adult in household^d^								
Less than college degree^e^	1023 (46.1)	448 (43.0)	660 (47.4)	544 (41.4)	877 (46.2)	1146 (41.1)	1144 (40.6)	5842 (43.7)
College graduate or higher	2315 (53.9)	960 (57.0)	1206 (52.6)	1063 (58.6)	1651 (53.8)	2531 (58.9)	2596 (59.4)	12 322 (56.3)
Maternal age at delivery, y^d^								
≤30	1674 (50.5)	693 (48.9)	889 (48.5)	729 (42.4)	1154 (44.9)	1587 (42.6)	1523 (43.9)	8249 (46.0)
>30	1635 (49.5)	699 (51.1)	963 (51.5)	862 (57.6)	1350 (55.1)	2036 (57.4)	2167 (56.1)	9712 (54.0)
Smoker in household^d^^,^^f^								
Yes	412 (16.6)	177 (11.7)	209 (10.6)	165 (12.4)	265 (11.9)	312 (10.4)	265 (7.8)	1805 (11.6)
No	2917 (83.4)	1206 (88.3)	1627 (89.4)	1413 (87.6)	2216 (88.1)	3282 (89.6)	3382 (92.2)	16 043 (88.4)
Child’s sex^g^								
Girl	1621 (49.5)	701 (47.5)	875 (47.9)	752 (48.2)	1206 (49.1)	1793 (48.9)	1829 (49.5)	8777 (48.6)
Boy	1737 (50.5)	707 (52.5)	991 (52.1)	855 (51.8)	1322 (50.9)	1884 (51.1)	1911 (50.5)	9407 (51.4)

^a^ Percentages are adjusted for National Survey of Children’s Health survey weights.

^b^ Race and ethnicity (child’s Hispanic origin had 0.3% missing cases and race had 1.7% missing cases) was imputed by the US Census Bureau using hot-deck imputation; Other includes Asian, American Indian or Alaska Native, Native Hawaiian and Other Pacific Islander, “some other race,” and “2 or more races.”

^c^ Family income (16.0%) was imputed by the US Census Bureau using sequential regression imputation methods.

^d^ Numbers vary slightly in the subgroups due to missing data.

^e^ Includes less than high school, high school, and some college or technical school.

^f^ Smoker in household was defined by answers to the question: “Does anyone living in your household use cigarettes, cigars, or pipe tobacco?”

^g^ Child’s sex (<0.1% missing) was imputed by the US Census Bureau using hot-deck imputation.

The overall prevalence of early introduction to complementary feeding was 10.7% (95% CI, 9.6%-11.8%) (Table 2). The percentage varied sociodemographically, with a substantially higher prevalence among Black children and children from households with low income, low education, and smoking. There were no significant changes in prevalence of early introduction of complementary foods between 2016 and 2022. However, a significant decrease in this practice was observed among White children, with a decrease from 11.2% (95% CI, 8.8%-13.5%) to 6.2% (95% CI, 4.6%-7.8%), with a −9.9% (95% CI, −15.0% to −4.4%) mean decrease per year cycle (*P* = .006 for trend).

**Table 2.** Trends in Prevalence of Early Introduction of Complementary Foods to US Infants Aged 4 Months to 1 Year From National Survey of Children’s Health, 2016 to 2022^a^

Early introduction of complementary foods^b^	2016		2017		2018		2019		2020		2021		2022		Overall		Mean annual % change (95% CI)	*P* value for trend
	No.	% (95% CI)	No.	% (95% CI)	No.	% (95% CI)	No.	% (95% CI)	No.	% (95% CI)	No.	% (95% CI)	No.	% (95% CI)	No.	% (95% CI)		
Total	347	13.4 (10.5 to 16.3)	127	8.7 (6.3 to 11.1)	197	14.5 (10.6 to 18.4)	144	10.8 (7.7 to 13.9)	186	8.7 (6.5 to 10.9)	295	10.3 (7.5 to 13.2)	249	8.3 (6.6 to 9.9)	1545	10.7 (9.6 to 11.8)	−6.4 (−13.7 to 1.6)	.09
Race and ethnicity^c^																		
Hispanic	38	11.3 (4.2 to 18.5)^d^	15	6.2 (1.5 to 10.9)^d^	30	16.6 (4.1 to 29.0)^d^	24	13.8 (4.8 to 22.8)^d^	32	9.3 (3.9 to 14.6)	55	16.8 (7.3 to 26.4)	42	7.9 (4.3 to 11.4)	236	11.7 (8.6 to 14.8)	−1.8 (−18.2 to 17.9)	.81
Non-Hispanic Black	31	27.5 (13.7 to 41.3)	13	18.6 (4.6 to 32.7)^d^	20	28.3 (15.3 to 41.3)	12	18.3 (6.2 to 30.3)^d^	18	16.0 (5.6 to 26.3)^d^	33	16.9 (8.4 to 25.4)	31	19.5 (10.9 to 28.2)	158	21.0 (16.4 to 25.5)	−6.7 (−14.5 to 1.8)	.10
Non-Hispanic White	236	11.2 (8.8 to 13.5)	81	8.8 (6.0 to 11.6)	123	11.9 (8.4 to 15.3)	92	8.0 (5.6 to 10.5)	103	7.0 (4.8 to 9.1)	162	6.3 (3.9 to 8.8)	126	6.2 (4.6 to 7.8)	923	8.5 (7.6 to 9.5)	−9.9 (−15.0 to −4.4)	.006^e^
Other	42	11.2 (6.2 to 16.2)	18	6.4 (1.8 to 11.0)^d^	24	9.5 (3.4 to 15.5)^d^	16	10.2 (2.9 to 17.4)^d^	33	7.6 (3.3 to 11.9)	45	9.5 (5.1 to 13.8)	50	7.5 (4.1 to 10.8)	228	8.6 (6.7 to 10.6)	−3.8 (−11.2 to 4.1)	.26
Family income, % federal poverty level^f^																		
<100	63	19.8 (12.2 to 27.4)	20	11.2 (4.1 to 18.3)^d^	39	22.8 (9.6 to 36.0)	24	14.9 (4.4 to 25.3)^d^	47	16.0 (9.0 to 23.1)	62	15.3 (8.5 to 22.2)	49	13.0 (8.1 to 17.9)	304	16.3 (13.0 to 19.7)	−5.1 (−12.8 to 3.3)	.17
100-199	58	14.4 (6.2 to 22.6)	31	11.5 (4.8 to 18.2)	46	16.8 (9.4 to 24.1)	34	14.9 (8.7 to 21.1)	40	12.3 (7.0 to 17.6)	58	10.5 (3.2 to 17.8)^d^	36	7.4 (4.2 to 10.6)	303	12.7 (10.2 to 15.3)	−9.1 (−21.7 to 5.5)	.21
200-399	105	9.5 (7.0 to 12.0)	34	8.2 (4.6 to 11.8)	51	9.3 (5.7 to 12.9)	51	12.0 (7.2 to 16.8)	50	6.2 (3.6 to 8.9)	84	11.7 (5.0 to 18.4)	75	8.2 (5.3 to 11.1)	450	9.2 (7.7 to 10.8)	−1.7 (−10.5 to 7.9)	.66
≥400	121	11.3 (8.0 to 14.5)	42	6.0 (3.5 to 8.5)	61	12.1 (7.0 to 17.2)	35	5.4 (2.6 to 8.1)	49	4.4 (1.6 to 7.3)^d^	91	6.6 (3.6 to 9.7)	89	6.4 (4.3 to 8.4)	488	7.3 (6.1 to 8.6)	−8.4 (−19.5 to 4.1)	.14
Highest education of adult in household^g^																		
Less than college degree^h^	168	18.6 (13.0 to 24.1)	60	11.4 (7.0 to 15.8)	95	19.1 (12.1 to 26.2)	78	16.3 (10.1 to 22.6)	95	14.0 (9.6 to 18.4)	129	16.8 (10.6 to 23.0)	109	11.3 (8.1 to 14.5)	734	15.4 (13.3 to 17.6)	−5.1 (−13.5 to 4.2)	.21
College graduate or higher	174	8.9 (6.6 to 11.2)	67	6.7 (4.1 to 9.3)	102	10.4 (6.9 to 13.8)	66	6.9 (4.2 to 9.5)	91	4.1 (2.7 to 5.6)	166	5.8 (4.2 to 7.5)	140	6.2 (4.5 to 7.8)	806	7.0 (6.1 to 7.9)	−7.4 (−16.5 to 2.7)	.11
Maternal age at delivery, y^g^																		
≤30	190	15.9 (11.1 to 20.8)	68	10.2 (6.3 to 14.0)	114	19.9 (12.9 to 26.8)	73	10.4 (6.8 to 14.0)	94	9.4 (6.6 to 12.2)	133	11.2 (6.3 to 16.1)	118	11.0 (8.0 to 13.9)	790	12.7 (10.9 to 14.5)	−6.1 (−16.5 to 5.5)	.22
>30	145	10.0 (7.0 to 13.1)	58	7.4 (4.5 to 10.2)	82	9.2 (6.0 to 12.4)	69	11.3 (6.6 to 16.0)	89	8.3 (4.9 to 11.6)	150	9.3 (5.8 to 12.9)	126	6.2 (4.5 to 8.0)	719	8.8 (7.5 to 10.1)	−5.2 (−12.8 to 3.1)	.16
Smoker in household^g^^,^^i^																		
Yes	63	18.4 (9.0 to 27.9)	27	10.7 (5.7 to 15.8)	35	13.8 (6.1 to 21.5)	19	16.6 (6.5 to 26.7)^d^	29	12.3 (5.5 to 19.1)	44	14.8 (4.1 to 25.4)^d^	36	21.4 (11.9 to 31.0)	253	15.3 (11.9 to 18.6)	5.2 (−6.6 to 18.5)	.32
No	278	12.3 (9.4 to 15.2)	98	8.5 (5.9 to 11.1)	158	14.1 (9.9 to 18.3)	120	8.7 (6.3 to 11.0)	149	8.2 (5.8 to 10.6)	246	10.1 (7.0 to 13.1)	203	6.7 (5.3 to 8.2)	1252	9.8 (8.7 to 10.9)	−7.9 (−15.9 to 0.8)	.06
Child’s sex^j^																		
Girl	147	14.3 (9.4 to 19.3)	60	7.0 (4.4 to 9.5)	84	10.9 (7.1 to 14.8)	61	12.4 (7.1 to 17.6)	83	8.5 (4.9 to 12.0)	129	7.5 (5.4 to 9.6)	126	8.4 (6.1 to 10.7)	690	9.8 (8.4 to 11.3)	−6.1 (−15.8 to 4.7)	.20
Boy	200	12.4 (9.4 to 15.5)	67	10.3 (6.4 to 14.2)	113	17.8 (11.4 to 24.2)	83	9.3 (6.0 to 12.7)	103	8.9 (6.2 to 11.6)	166	13.1 (8.0 to 18.2)	123	8.1 (5.8 to 10.4)	855	11.5 (9.9 to 13.1)	−6.0 (−15.5 to 4.6)	.20

^a^ All estimates, except sample sizes, are weighted.

^b^ Early introduction of complementary foods was defined as first fed anything other than breast milk or formula at younger than 4 months, including water, juice, cow’s milk, sugar water, baby food, or anything else.

^c^ Race and ethnicity (child’s Hispanic origin had 0.3% missing cases and race had 1.7% missing cases) was imputed by the US Census Bureau using hot-deck imputation; Other includes Asian, American Indian or Alaska Native, Native Hawaiian and Other Pacific Islander, “some other race,” and “2 or more races.”

^d^ Point estimates may be unreliable (95% CI width >1.2 times the estimate).

^e^ *P* < .01.

^f^ Family income (16.0%) was imputed by the US Census Bureau using sequential regression imputation methods.

^g^ Numbers vary slightly in the subgroups due to missing data.

^h^ Includes less than high school, high school, and some college or technical school.

^i^ Smoker in household was defined by answers to the question: “Does anyone living in your household use cigarettes, cigars, or pipe tobacco?”

^j^ Child’s sex (<0.1% missing) was imputed by the US Census Bureau using hot-deck imputation.

## Discussion

We found that 1 in 10 US children was introduced to complementary foods before 4 months; this was more common among children from households with lower socioeconomic status, suggesting a similar sociodemographic pattern but lower prevalence of early complementary food introduction compared with previous studies.^4,5^ In this study, a downward trend became apparent across most groups, but was statistically significant only among White children. The findings suggest more education is needed to reduce early complementary feeding practices among US women of racial and ethnic minority groups. Limitations include that the data do not allow for causal inferences about the effects of other determinants on the introduction of complementary foods. To promote adherence to guidelines for introducing complementary foods, health care professionals may need to assess and address maternal beliefs about feeding infants younger than 6 months old substances other than breast milk or formula.

## Notes

Supplement.
Data Sharing Statement
