# Supplementary material for: Trends in Prevalence of Early Introduction of Complementary Foods to US Children, 2016 to 2022
Source: JAMA Netw Open. 2024 Oct 11;7(10):e2440255. doi: 10.1001/jamanetworkopen.2024.40255 (PMC11581586; doi:10.1001/jamanetworkopen.2024.40255)
Supplement: Supplement. — Data Sharing Statement [file jamanetwopen-e2440255-s001.pdf]

Data Sharing Statement

Ding. Trends in Prevalence of Early Introduction of Complementary Foods to US Children, 2016 to 2022. *JAMA Netw Open*. Published October 11, 2024.
doi:10.1001/jamanetworkopen.2024.40255

Data
Data available: Yes
Data types: Deidentified participant data
How to access data: Data sharing will be available from YZ upon a reasonable request. Electronic address: zhangyongjun@sjtu.edu.cn.
When available: beginning date: 12-01-2024, end date: 12-01-2025

Supporting Documents
Document types: Statistical/analytic code
How to access documents: Data sharing will be available from YZ upon a reasonable request. Electronic address: zhangyongjun@sjtu.edu.cn.
When available: beginning date: 12-01-2024, end date: 12-01-2025

Additional Information
Who can access the data: Data sharing will be available from YZ upon a reasonable request. Electronic address: zhangyongjun@sjtu.edu.cn.
Types of analyses: Data sharing will be available from YZ upon a reasonable request. Electronic address: zhangyongjun@sjtu.edu.cn.
Mechanisms of data availability: Data sharing will be available from YZ upon a reasonable request. Electronic address: zhangyongjun@sjtu.edu.cn.
Any additional restrictions: Data sharing will be available from YZ upon a reasonable request. Electronic address: zhangyongjun@sjtu.edu.cn.
